# Development and Validation of a Resilience Scale for Nursing Home Staff

**DOI:** 10.1111/opn.70069

**Published:** 2026-02-12

**Authors:** Sung Ok Chang, Eun Young Kim

**Affiliations:** ^1^ College of Nursing Korea University Seoul Republic of Korea; ^2^ School of Nursing Soonchunhyang University Cheonan Republic of Korea

**Keywords:** geriatric nursing, long‐term care, nursing homes, nursing staff, resilience, scale development

## Abstract

**Introduction:**

Nursing home staff experience sustained emotional and physical stress. Existing resilience scales, developed for the general population or hospital‐based, do not capture the specific demands of nursing home care, including chronic staffing shortages, emotional labour and end‐of‐life care complexities. This study developed and validated a resilience scale tailored to nursing home staff.

**Methods:**

Following DeVellis's scale development guidelines, 26 preliminary items were generated and refined to 21 through expert content validation. Data were collected from 302 nursing home staff (registered nurses, nurse assistants and care workers) employed at 25 nursing homes in Seoul, South Korea, between 1 July and 31 August, 2023. The validity and reliability were examined using exploratory and confirmatory factor analyses.

**Results:**

The final scale comprised 17 items across four factors: Emotional Self‐Regulation and Self‐Efficacy; Meaning and Personal Growth through Caregiving; Support‐Seeking and Meaning‐Based Coping; and Professional Growth Orientation. The model demonstrated good fit (CMIN/DF = 1.76, CFI = 0.95, RMSEA = 0.07; SRMR = 0.08), strong internal consistency (*α* = 0.88) and significant correlation with CD‐RISC (*r* = 0.68, *p* < 0.001).

**Conclusion:**

This validated context‐specific scale reflects the unique resilience of nursing home practices and offers a tool for assessing and strengthening psychological resources among staff in nursing home settings.

## Summary Statement of Implications for Practice

**What does this research add to existing knowledge in gerontology?:**

- Develops and validates a resilience scale designed for nursing home staff, addressing unique nursing home care demands not captured by existing general or hospital‐based tools.
- Reconceptualises resilience as a multifaceted construct shaped by professional identity, emotional self‐regulation, meaning‐making in caregiving and context‐specific coping strategies in caring for older adults.

**What are the implications of this new knowledge for nursing care for and with older adults?:**

- Enables early identification of nursing home staff at risk of burnout or moral distress, facilitating timely targeted interventions to strengthen resilience and well‐being.
- Supports improvements in the continuity of care, emotional presence and quality of life for older residents, particularly those living with dementia or at end‐of‐life.

**How could the findings be used to influence practice, education, research, and policy?:**

- Provides policymakers with a reliable tool for assessing and monitoring resilience among nursing home staff, guiding targeted resource allocation and workforce planning.
- Offers researchers and educators a validated instrument to design and evaluate resilience‐enhancement programmes and explore links between resilience, job satisfaction, retention and care quality.

## 1 Introduction

The global population is rapidly aging, leading to an increased burden of age‐related diseases such as dementia and a growing demand for complex long‐term care (Xiaopeng et al. 2025). Consequently, nursing homes (NHs) have become essential institutions where a diverse nursing staff, including registered nurses (RNs), nurse assistants (NAs) and care workers (CWs), provide critical clinical and psychosocial support (National Health Insurance Service [NHIS] 2024; Zhang et al. 2023).

Despite their crucial role, NH staff are frequently exposed to significant psychological and physical stressors. These include a high patient load, emotionally taxing care tasks, exposure to patient suffering and death, and inadequate staffing and compensation (Chang and Kim 2022; Zhang et al. 2023). In addition to heavy workloads and systemic challenges, nursing staff in NHs are inevitably exposed to emotional and physical exhaustion as they continuously manage the behavioural and psychological symptoms of dementia, advanced aging and progressive functional decline (Costello et al. 2019). Moreover, caring for residents with dementia in such settings often involves ethically challenging situations, leading to moral distress among nursing staff (Kim et al. 2025).

These diverse negative experiences among nursing staff in NHs compromise the quality of care and contribute to staff turnover, increasing social and economic costs (Pradhan et al. 2025; Shen et al. 2023). Ultimately, these challenges undermine the quality of care provided to residents (Kowalczuk et al. 2020). Therefore, it is imperative to explore the difficulties nursing staff face in NHs and develop protective strategies to mitigate their adverse effects (McKenna et al. 2022). In this environment, resilience serves as an essential psychological resource and a protective factor against burnout, emotional exhaustion and turnover intention, which are often precipitated by the persistent psychological, physical and ethical demands of NH care (Baminiwatta et al. 2025; Chang and Kim 2022). Resilience, which is commonly defined as the capacity to recover and adapt positively in the face of adversity or stress, has been shown to mitigate emotional exhaustion, reduce turnover intention and enhance job satisfaction and performance among healthcare professionals (Gao et al. 2017; Wei et al. 2019). Several studies have demonstrated that resilience in nursing personnel is associated with better patient care outcomes, increased organisational commitment and reduced burnout (Nantsupawat et al. 2024; Poku et al. 2025). Resilience requires a context‐sensitive perspective and identifying its attributes necessitates an approach that is appropriate for the cultural setting in which such concept is situated (Ungar and Theron 2020). However, most existing resilience measurement scales, such as the Connor–Davidson Resilience Scale (CD‐RISC; Connor and Davidson 2003), Brief Resilience Scale (BRS; Smith et al. 2008) and Resilience Scale for Adults (RSA; Friborg et al. 2003)—were initially developed for the general population or acute care nurses in hospital settings. However, these scales do not sufficiently reflect the distinct structural, emotional and interpersonal stressors of NHs. In particular, the interdependent nature of work in NHs, where RNs, NAs and CWs operate in overlapping roles under fluid hierarchies, requires a broader and more nuanced conceptualisation of resilience.

Therefore, this study aims to develop and validate a resilience measurement scale tailored to the unique characteristics of NH care personnel. This enables the early identification of nursing staff (RNs, NAs and CWs) at risk of burnout or turnover. Furthermore, it supports targeted resilience‐building training and contributes empirical data for implementing staff development and organisational support programmes to sustain workforce well‐being and improve care quality.

### 1.1 Study Objective

This study aimed to develop a psychometrically sound resilience scale for nursing staff in NHs and to examine its validity and reliability.

## 2 Methods

### 2.1 Research Design

This study was conducted to develop and evaluate a resilience measurement scale for nursing staff working in NHs following the guidelines proposed by DeVellis (2023) for scale construction.

### 2.2 Ethical Considerations and Data Collection

Before the commencement of the study, ethical approval was obtained from the Institutional Review Board (IRB) of Soonchunhyang University (IRB No. 1040875–202,212‐SB‐133). Data were collected between 1 July and 30 September, 2023. Recruitment notices were posted for 2 weeks on both online and offline bulletin boards of the 25 NHs. Participants expressed their willingness and consent to participate after reviewing the purpose and procedures of the study. Data collection was conducted using two methods: face‐to‐face surveys administered directly by the researcher, and self‐administered online surveys via Google Forms, which were accessed through the links sent to participants' smartphones.

### 2.3 Research Procedure

An overview of the study timeline is as follows:

Phase 1. Scale Development: (Jan–Mar 2023): Preliminary item generation and expert content validity assessment.

Phase 2. Scale Validation: (Jul 2023–Feb 2024): Main data collection and comprehensive data analysis.

Each of these phases is described in detail in the following sections.

### 2.4 Phase 1: Scale Development

#### 2.4.1 Preliminary Item Generation

The preliminary items were generated by two researchers based on the conceptual framework established in their earlier conceptual development study (Chang and Kim 2022). Each attribute identified in the framework—optimism, patience, mindfulness, supportive relationships, available resources, work–life boundary setting, self‐development and growth—was reviewed, and items were written to reflect its conceptual meaning. The number of items per attribute was not predetermined. Rather, the items were created according to the conceptual breadth and the characteristics of each attribute. This resulted in natural variations across categories. Through this process, 26 preliminary items were developed: five for optimism, two for patience, three for mindfulness, five for supportive relationships, two for available resources, three for work–life boundary setting, four for self‐development and two for growth. All items were rated on a five‐point Likert scale.

#### 2.4.2 Expert Content Validity Assessment

The preliminary items were evaluated for content validity by a panel of 10 experts, comprising eight administrators with over 10 years of experience in NHs and two nursing professors specialising in geriatric care. Content validity testing was conducted in two rounds using the item‐content validity index (I‐CVI), with a criterion of I‐CVI ≥ 0.80 considered acceptable. The I‐CVI was calculated using a four‐point Likert scale. Expert feedback was collected for items requiring addition, revision or removal. Based on this feedback, items with overlapping meanings were integrated, and ambiguous expressions were revised and refined for clarity.

### 2.5 Phase 2: Scale Validation

#### 2.5.1 Instrument Evaluation

##### 2.5.1.1 Participants

Based on the average staffing structure of NHs in South Korea and an expected response rate of approximately 60%–70%, it was estimated that participants would need to be recruited from approximately 20 to 25 NHs to achieve a target sample size of 300. The researcher identified the target NHs through Internet searches and contacted administrators to request their cooperation. Subsequently, recruitment announcements were posted on the bulletin boards of the 25 facilities that consented to participate. The NHs that agreed to participate in the study were located in Seoul, South Korea and varied in capacity (ranging from 50 to 350 residents). The announcements included the research title, purpose, methodology, inclusion and exclusion criteria, potential risks and benefits of participation, and the contact information of the research team.

Voluntary participants were screened to determine their eligibility based on inclusion and exclusion criteria. Those who met the criteria and provided informed consent after being fully informed of the purpose and procedures of the study were selected as research participants.

The inclusion criterion was nursing staff (RNs, NAs and CWs) currently working in NHs who agreed to participate in the study. The exclusion criterion was nursing staff with less than 12 months of work experience in NHs. This criterion was based on prior literature suggesting that adequate expertise is necessary to meaningfully respond to questions related to resilience, as individuals with less than 12 months of experience are typically considered to have the lowest level of clinical competence (Aziznejadroshan et al. 2024). Data collection was conducted either through researcher‐administered face‐to‐face sessions or self‐administered online surveys via Google Forms links sent to the participants' smartphones. Of the 310 questionnaires collected, 302 were included in the final analysis after excluding duplicate responses. Participants reported that the 17‐item scale was concise and easy to understand, with an average completion time of less than 10 min.

##### 2.5.1.2 Data Analysis

To ensure the structural integrity of the scale, the total sample of 302 participants was randomly divided into two independent subsamples. Each participant was assigned a random number using a computer‐generated sequence, and the sample was divided into two groups (*n* = 151 each) for exploratory and confirmatory analyses. Based on the evidence that conducting both exploratory factor analysis (EFA) and confirmatory factor analysis (CFA) on the same sample may compromise construct validity (Hinkin 1998), the first subsample (Time 1 = 151) was used for EFA to identify the initial factor structure, and the second subsample (Time 2 = 151) was used for CFA to validate the model fit.

Validity and reliability were assessed using SPSS Windows software (version 26.0) and R software (version 4.4.1) with the lavaan package (version 0.6–21). To account for the ordinal nature of the Likert scale data, a CFA was performed using the Weighted Least Squares Mean and Variance adjusted (WLSMV) estimator based on polychoric correlation matrices, as recommended for robust parameter estimation.

Before evaluating construct validity, item analysis was conducted to determine the appropriateness of the collected data. This included analysing the mean, standard deviation, skewness and kurtosis of each item. Item‐total correlation coefficients were examined to evaluate the contribution of each item.

Construct validity, including convergent and discriminant validity, was assessed using both EFA and CFA.

The initial factor structure was explored using EFA with principal component analysis and varimax rotation. Items were retained if communalities were ≥ 0.50 and factor loadings were ≥ 0.40 on primary factors (Acar Güvendir and Özer Özkan 2022). Items with loadings ≥ 0.32 on more than one factor and less than 0.10 difference between loadings were removed to ensure a clean factor structure (Acar Güvendir and Özer Özkan 2022). Following the EFA, a CFA was conducted to validate the scale's structural integrity. Model fit in CFA was evaluated using several fit indices, including the chi‐square test (*χ* ^2^), the chi‐square minimum to degrees of freedom ratio (CMIN/DF or *χ* ^2^/df), the standardised root mean square residual (SRMR), the root mean square error of approximation (RMSEA), the Tucker–Lewis index (TLI) and the comparative fit index (CFI). Given that the chi‐square test is sensitive to model complexity and sample size, the model fit was primarily interpreted using CMIN/DF. A CMIN/DF ≤ 3, SRMR ≤ 0.08, RMSEA ≤ 0.06, and CFI and TLI ≥ 0.95 were considered indicative of a good model fit (Howard et al. 2025).

Convergent validity, which examines the consistency among items within each construct, was assessed using standardised factor loadings, average variance extracted (AVE) and construct reliability (CR) derived from a confirmatory factor analysis.

Discriminant validity, which verifies the distinction between constructs, was evaluated by comparing the AVE values of each construct with the squared correlations between the latent variables.

For reliability, internal consistency was primarily evaluated using the CR and AVE values derived from the CFA, whereas test–retest reliability was examined using Pearson's correlation to ensure temporal stability over a two‐week interval.

## 3 Results

### 3.1 Scale Development

#### 3.1.1 Preliminary Item Development and Expert Content Validity Assessment

Initially, a total of 26 preliminary items were generated based on the eight attributes identified in the conceptual framework (Chang and Kim 2022). Based on the I‐CVI results, five items were removed because their scores were below the threshold of 0.80. The excluded items were, ‘When I am going through a hard time, I gain strength by recalling moments of joy and happiness’. ‘I believe everything depends on mindset, and if I care for my mind, things will work out’. ‘I trust and rely on my coworkers, who understand my situation better than anyone’. ‘Instead of bottling everything up, I try to determine what national, social, and interpersonal resources are available to support me’. ‘After work, I try not to think about work at all’.

Additionally, six items were modified according to expert recommendations to improve semantic clarity and ensure alignment with the intended conceptual domains. In the second round of validation, all the remaining items achieved an I‐CVI of 1.00, confirming their content validity. As a result, 21 items were finalised.

### 3.2 Scale Validation

#### 3.2.1 General Characteristics of the Participants

Among the 302 participants, 90.02% were female (Table 1). The age of the participants ranged from 20 to 60 years old, with a mean age of 50.37 years. Regarding educational attainment, the majority (41.72%) had completed high school, followed by those with a bachelor's, associate's or master's degree. Their clinical experience was 10.7 years, and their average experience with NHs was 5.75 years. Regarding job roles, 53.3% were CWs, 33.44% RNs and 13.25% NAs. The overall mean resilience score of the 302 participants was 54.40 (SD = 7.36). The mean resilience scores by job category were 54.75 (SD = 7.25) for care workers, 53.56 (SD = 7.49) for nurses and 55.09 (SD = 7.64) for nurse assistants, on a scale ranging from 0 to 68. Out of all the respondents, 158 (52.3%) completed the online survey and 144 (47.7%) completed the face‐to‐face survey.

**TABLE 1:** General characteristics of participants (*N* = 302).

Characteristics	Category	*N* (%)	M ± SD
Sex	Men	9 (2.98)	
	Women	293 (97.02)	
Age (year)	20–29	18 (5.96)	50.37 ± 10.88
	30–39	43 (14.24)	
	40–49	53 (17.55)	
	50–59	128 (42.38)	
	60–65	60 (19.87)	
Final education level	High school	126 (41.72)	
	Associate degree	65 (21.52)	
	Bachelor's degree	97 (32.12)	
	Master's degree	14 (4.64)	
Overall experience (year)	1–5	106 (35.10)	10.69 ± 8.99
	6–10	49 (16.23)	
	11–15	59 (19.54)	
	16–20	38 (12.58)	
	21–25	20 (6.62)	
	26–30	15 (4.97)	
	31–35	15 (4.97)	
Nursing home experience	1–5	167 (55.30)	5.75 ± 5.06
	6–10	76 (25.17)	
	11–15	40 (13.25)	
	16–20	16 (5.30)	
	21–25	3 (0.99)	
Job title	Care worker	161 (53.31)	
	Nurse	101 (33.44)	
	Nurse assistant	40 (13.25)	
Resilience score (0–68)	Care worker		54.75 ± 7.25
	Nurse		53.56 ± 7.49
	Nurse assistant		55.09 ± 7.64
	Total		54.40 ± 7.36

#### 3.2.2 Item Analysis

The skewness values for the 21 items ranged from –0.84 to 0.98, and the kurtosis values ranged from −0.40 to 1.02, indicating that all items fell within acceptable ranges for normality (i.e., |skewness| < 2 and |kurtosis| < 7) (Kim 2013). The mean scores of the preliminary items ranged from 2.49 to 3.49, with standard deviations between 0.60 and 0.97 (Table 2). Item‐total correlation analysis was conducted to examine the internal consistency of the scale. Two items (Items 9 and 11) showed relatively low item‐total correlation coefficients of 0.30 and 0.28, respectively, suggesting limited contribution to the overall scale. Furthermore, the communality values of Items 9, 11 and 12 were below 0.50, indicating a low shared variance with the underlying construct. Consequently, these three items were removed, and a final set of 18 items was retained for further analysis.

**TABLE 2:** Analysis of the 21 items.

No	Item contents	M ± SD	Item‐total correlation	Communality
1	I believe that caring for older adults in nursing homes is a significant job.	3.49 ± 0.60	0.53	0.55
2	I am satisfied with my life, where I strive harder than anyone else.	3.19 ± 0.71	0.56	0.65
3	I believe I can overcome challenges and exhaustion on my own.	3.19 ± 0.69	0.60	0.55
4	I think the work I am doing now is something I can do well.	3.25 ± 0.64	0.57	0.52
5	Even when the work is difficult, I believe I can do it independently.	3.11 ± 0.64	0.59	0.61
6	In difficult situations, I remind myself that this moment will pass.	3.10 ± 0.70	0.55	0.50
7	I focus on myself and try to manage my inner thoughts and emotions.	3.14 ± 0.72	0.59	0.59
8	I have my own ways to soothe my mind in difficult situations.	2.96 ± 0.76	0.65	0.61
9	When work gets tough, I rely on family, friends, and coworkers to get through it.	2.56 ± 0.94	0.30	0.41
10	Whenever I feel exhausted while working in the nursing home, I think of the residents who need my help, and it gives me strength.	2.73 ± 0.74	0.63	0.51
11	Thinking of my family waiting for me at home gives me energy.	3.12 ± 0.81	0.28	0.48
12	I believe that my manager will always be there to support me if I ask for help.	2.78 ± 0.80	0.42	0.43
13	When I need help while working in the nursing home, I have access to support resources such as counselling.	2.49 ± 0.93	0.53	0.60
14	I recharge myself by taking enough rest and enjoying my hobbies on days off or after work.	2.93 ± 0.97	0.59	0.83
15	I believe that getting enough rest is a reward for working hard.	3.20 ± 0.74	0.49	0.84
16	I constantly learn and study to provide better care for the nursing home residents.	2.89 ± 0.68	0.48	0.74
17	I strive to live a better life than I do now.	3.20 ± 0.71	0.56	0.74
18	I set goals for my life and work hard to achieve them.	3.09 ± 0.74	0.55	0.73
19	I want to become an expert in geriatric nursing and provide professional care.	3.16 ± 0.72	0.56	0.57
20	Caring for nursing home residents makes me feel that I have grown as a person.	3.09 ± 0.78	0.66	0.67
21	The diverse experiences I have had in the nursing home have taught me many valuable lessons in life.	3.25 ± 0.71	0.50	0.67

#### 3.2.3 Exploratory Factor Analysis

To assess the suitability of the data for factor analysis, the Kaiser–Meyer–Olkin (KMO) measure of sampling adequacy and Bartlett's test of sphericity were used. The KMO value was 0.81, and Bartlett's test was significant (*χ* ^2^ = 384.51, *p* < 0.001) (Table 3), indicating that the data were appropriate for exploratory factor analysis (EFA) (Sigudla and Maritz 2023).

**TABLE 3:** Factor loadings of items per factor, eigenvalues and reliability of the final 17 items.

Factor name	Item No.	Factor loading			
		1	2	3	4
1. Emotional self‐regulation and self‐efficacy	7	0.76	0.20	0.19	−0.03
	5	0.74	0.05	0.09	0.20
	4	0.72	0.07	0.22	0.29
	3	0.70	0.02	0.02	0.18
	2	0.68	0.23	0.28	0.04
	6	0.66	−0.03	0.14	0.30
	8	0.64	0.08	0.07	0.21
2. Meaning and personal growth through caregiving	20	0.19	0.88	0.20	0.08
	21	0.22	0.84	0.08	0.16
	1	0.06	0.62	0.21	0.06
3. Support‐seeking and meaning‐ based coping	10	0.27	0.13	0.78	0.26
	14	0.11	0.06	0.61	0.05
	13	0.17	−0.02	0.55	0.12
4. Professional growth orientation	19	0.04	0.25	0.13	0.81
	18	0.23	0.29	0.03	0.70
	16	0.10	0.19	0.02	0.65
	17	0.07	0.08	0.06	0.64
Number of items		7	3	3	4
Eigen value		3.42	1.86	1.30	1.99
Explained variance (%)		20.13	10.95	7.64	11.68
Accumulative variance (%)		20.13	31.08	38.72	50.40
Reliability: Cronbach's *α*				0.88	
KMO	0.81				
Bartlett's Test of sphericity	Approximate Chi‐Square: 384.51, df = 136, *p* < 0.001				

During the factor purification process, item R15 was removed because it did not load significantly on any factor (loading < 0.40). Consequently, 17 items were retained in the final factor structure.

The first factor, Emotional Self‐Regulation and Self‐Efficacy, consisted of seven items (Items 7, 5, 4, 3, 2, 6 and 8) that captured the ability to manage emotional responses and maintain confidence in one's capacity to overcome challenges. These include cognitive reframing, emotional endurance and a sense of control. The seven items included in this factor had loadings ranging from 0.64 to 0.76. The eigenvalue was 3.42, accounting for 20.13% of total variance.

The second factor, Meaning and Personal Growth through Caregiving (Items 20, 21 and 1), reflected the sense of meaning and personal development derived from the caregiving experience. This illustrates how the nursing staff find value in their work and perceive caregiving as a source of personal growth. The three items included in this factor had loadings ranging from 0.62 to 0.88. The eigenvalue was 1.86, accounting for 10.95% of total variance.

The third actor, Support‐Seeking and Meaning‐Based Coping, comprised Items 10, 14 and 13 and describes the use of emotional soothing, external support systems and meaning‐based strategies to cope with occupational stress. The three items included in this factor have loadings ranging from 0.55 to 0.78. The eigenvalue was 1.30, which accounted for 7.64% of the total variance. These findings highlight the role of interpersonal and intrapersonal resources in building and maintaining resilience.

The fourth factor, Professional Growth Orientation, included four items (Items 19, 18, 16 and 17) that reflected individuals' motivation to improve professionalism, set goals and strive for better performance in caregiving. This factor represents a forward‐looking attitude towards self‐development and specialisation in geriatric nursing. The four items included in this factor had loadings ranging from 0.64 to 0.81. The eigenvalue of this factor was 1.99, accounting for 11.68% of total variance.

#### 3.2.4 Confirmatory Factor Analysis

To evaluate the adequacy of the four‐factor structure identified through exploratory factor analysis (EFA), a confirmatory factor analysis (CFA) was conducted on the final set of 17 items.

##### 3.2.4.1 Model Fit

The results of the confirmatory factor analysis (CFA) indicated that the four‐factor model demonstrated an acceptable fit to the data: *χ* ^2^(113) = 198.95, *p* < 0.001; CMIN/DF: 1.76; CFI = 0.95; TLI = 0.95; NFI = 0.94; RMSEA = 0.07; SRMR = 0.08 (Table 4). These indices suggest that the model has good construct validity and adequately represents the underlying factor structure identified in EFA.

**TABLE 4:** Constructing the validity of the resilience scale for nursing staff in nursing homes.

Factor	Item.	Standardised estimate	SE	Squared correlation (Φ)				AVE	CR
				1	2	3	4		
1	7	0.76	0.08	1				0.49	0.87
	5	0.74	0.10						
	4	0.72	0.09						
	3	0.70	0.06						
	2	0.68	0.09						
	6	0.66	0.09						
	8	0.64	0.09						
	20	0.88	0.15	0.36	1			0.62	0.83
2	21	0.84	0.16						
	1	0.62	0.22						
	10	0.78	0.28	0.32	0.41	1		0.43	0.69
3	14	0.61	0.10						
	13	0.55	0.10						
	19	0.81	0.13	0.37	0.49	0.42	1	0.50	0.80
	18	0.70	0.11						
4	16	0.65	0.11						
	17	0.64	0.10						
Fitness index		*χ* ^2^(113) = 198.95, *p* < 0.001; CMIN/DF: 1.76, CFI = 0.95, TLI = 0.95, NFI = 0.94, RMSEA = 0.07; SRMR = 0.08							

##### 3.2.4.2 Convergent Validity and Discriminant Validity

Convergent validity was evaluated based on the criterion that the Average Variance Extracted (AVE) for each of the four factors should ideally exceed 0.50, and the Construct Reliability (CR) should be greater than 0.70 (Cheung et al. 2024). While a CR between 0.60 and 0.70 is considered acceptable (Cheung et al. 2024), the CR values in this study ranged from 0.69 to 0.87, and AVE values ranged from 0.43 to 0.62. Although the AVE for one factor was slightly below the 0.50 threshold, the convergent validity was deemed acceptable because the corresponding CR exceeded the recommended level, confirming that the scale demonstrated adequate convergent validity (Table 4).

Discriminant validity was assessed using the Fornell and Larcker (1981) criterion, which states that each construct's average variance extracted (AVE) should exceed the squared correlations between that construct and all other constructs.

All the four factors in this study met this criterion. Specifically, the AVE for Factor 1 (0.49) was greater than its squared correlations with other factors (0.36, 0.32, 0.37); Factor 2's AVE (0.62) exceeded its squared correlations (0.36, 0.41, 0.49); Factor 3's AVE (0.43) was higher than the squared correlations (0.32, 0.41, 0.42); and Factor 4's AVE (0.50) surpassed the squared correlations (0.37, 0.49, 0.42). These results provide evidence of adequate discriminant validity among the four factors.

#### 3.2.5 Concurrent Validity

A correlational analysis was conducted using the CD‐RISC, a well‐established measure of resilience, to assess the concurrent validity of the newly developed scale (Table 5). The results indicated a statistically significant positive correlation between the scores on the two scales (*r* = 0.68, *p* < 0.001), thus supporting the concurrent validity of the scale. Additionally, each of the four subscales demonstrated significant positive correlations with the CD‐RISC: Factor 1 (*r* = 0.61), Factor 2 (*r* = 0.65), Factor 3 (*r* = 0.59) and Factor 4 (*r* = 0.62), all *p* < 0.001. These findings suggest that the overall scale and its subdimensions are meaningfully associated with general resilience, providing further evidence for its validity.

**TABLE 5:** Criterion validity test with the CD‐RISC (*N* = 302).

	F1	F2	F3	F4	Total
CD‐RISC(25)	0.61 (< 0.001)	0.65 (< 0.001)	0.59 (< 0.001)	0.62 (< 0.001)	0.68 (< 0.001)

Abbreviation: F, factor.

#### 3.2.6 Assessment of Reliability

The internal consistency of the final 17‐item scale was evaluated using both Cronbach's alpha and the Composite Reliability (CR) derived from the CFA. The Cronbach's alpha for the entire scale was 0.88, indicating high internal reliability. For the four subscales, the CR values, which provided more accurate reliability estimates for latent constructs, were as follows: Factor 1 = 0.87, Factor 2 = 0.83, Factor 3 = 0.69 and Factor 4 = 0.81. The test–retest reliability was examined to evaluate the stability of the scale over time. The results showed a significant correlation of 0.81 ($*p* < 0.001$) between the first and second administrations with a two‐week interval, confirming the temporal stability of the scale. These findings demonstrated that the scale had robust internal consistency and overall reliability.

These findings demonstrate that the scale has acceptable internal consistency and overall reliability.

#### 3.2.7 Finalisation of the Instrument

Based on the results of the validity and reliability analyses, the resilience scale for NH staff was finalised with 17 items comprising four factors: Emotional Self‐Regulation and Self‐Efficacy (seven items), Meaning and Personal Growth through Caregiving (three items), Support‐Seeking and Meaning‐Based Coping (three items) and Professional Growth Orientation (four items). Each item is rated on a five‐point Likert scale (0–4), and the total score ranges from 0 to 68, with higher scores indicating greater resilience.

## 4 Discussion

NH staff provide physical, cognitive and emotional support in complex situations such as dementia and end‐of‐life care (Akunor et al. 2022). These demands increase the risk of emotional exhaustion and job stress, underscoring the importance of resilience as a key resource for maintaining well‐being and quality of care. Therefore, resilience must be understood within the specific context of NH care, highlighting the need for measurement tools that reflect the reality of geriatric nursing. Furthermore, by employing the WLSMV estimator, this study ensured psychometric rigour by accounting for the ordinal nature of the Likert scale data, thereby providing more robust and accurate parameter estimates than traditional maximum likelihood methods.

The final scale is comprised 17 items across four factors: Emotional Self‐Regulation and Self‐Efficacy, Meaning and Personal Growth through Caregiving, Support‐Seeking and Meaning‐Based Coping and Professional Growth Orientation. These dimensions align with previous findings that resilience develops through processes such as self‐reflection, meaning‐making and personal growth (Autio et al. 2024; Wang 2023). Compared to general resilience measures such as the CD‐RISC and RSA (Connor and Davidson 2003; Friborg et al. 2003), this scale captures the psychosocial, ethical and organisational challenges unique to NH practice. The factor structure reflects resilience as a multifaceted construct shaped by professional identity, emotional labour and supportive resources. Notably, the ‘Emotional Self‐Regulation and Self‐Efficacy’ factor emerged as the most substantial component of the scale, comprising seven items. This finding reinforces the notion that the ability to manage complex emotions and maintain a belief in one's professional competence is the core mechanism of resilience among NH staff. Given the high emotional labour inherent in geriatric care, these results suggest that internal psychological resources for self‐regulation are fundamental to sustaining caregiving quality and psychological well‐being. In particular, the inclusion of ‘Meaning and Personal Growth through Caregiving’ and ‘Support‐Seeking and Meaning‐Based Coping’ highlights coping processes that are especially relevant in environments characterised by repeated loss and interdisciplinary ambiguity.

The emergence of the ‘Support‐Seeking and Meaning‐Based Coping’ factor further distinguishes this scale from more traditional resilience measures. In NHs, where interdisciplinary collaborations and moral dilemmas frequently arise, the ability to derive meaning from one's role and seek emotional or social support is critical (Amin et al. 2025; Umubyeyi et al. 2024). These findings align with those of previous qualitative studies that reported that geriatric nurses and care workers often experience emotional fatigue due to repeated patient loss and ambiguous team roles. Therefore, they require context‐specific coping strategies (Dong et al. 2025; Zhang et al. 2023).

There were several considerations for research participation. In this study, more than half the participants were care workers, followed by registered nurses and nurse assistants, reflecting the typical staffing structure of Korean NHs. Although the scale showed a strong psychometric performance across all participants, the predominance of care workers may have affected the expression of certain resilience attributes. As care workers often engage in extensive daily support, emotional labour and less formal clinical training, their resilience may be shaped more by meaning‐making, emotion regulation and support‐seeking than by clinical decision‐making or professional development.

Thus, while the scale captures shared resilience components across roles, some subscales—such as Professional Growth Orientation and Emotional Self‐Regulation and Self‐Efficacy—may function differently depending on the training and scope of practice. Future research should include subgroup or multigroup analyses to examine measurement invariance across RNs, NAs and care workers, and determine whether role‐specific refinement is needed.

These role‐related considerations have implications for scale use in countries with different staffing structures. The staffing profile in this study differs from that in countries such as the United States and Canada, where NAs and licenced practical nurses constitute the majority of direct care personnel and typically assume broader clinical responsibilities. These differences may influence how staff perceive and operationalise resilience.

Although several items in the scale were intentionally designed to be context‐agnostic and applicable across various levels of training, cross‐cultural and cross‐role validation is still warranted. Researchers in other countries should examine factor stability, measurement invariance and potential cultural differences in resilience expression, particularly within hierarchical or clinical intensive care models. Such validation is essential before widespread international adoption.

Extending this consideration beyond national contexts, resilience may manifest differently across the long‐term care sector. Although the scale was developed for NH settings, its core attributes—emotional regulation, meaning‐making, professional growth and support‐seeking—are also relevant to assisted living, home care and community‐based dementia care. Variations in care intensity, team structure and exposure to end‐of‐life care may also influence resilience. Therefore, the application of the scale beyond NHs should be done cautiously, and sector‐specific validation studies are needed to determine whether the four‐factor structure remains stable in these environments.

The findings of the present study have several implications. The developed scale offers a robust and context‐specific tool for assessing resilience among the NH staff, enabling administrators and policymakers to better understand the psychosocial resources available to their workforce. The scale can help identify staff members who may benefit from additional support or resilience‐enhancing initiatives and guide the development of targeted educational and training programmes. While resilience is widely recognised as an important factor influencing well‐being and workforce stability, the present findings highlight the value of systematic resilience assessments in NH settings. Future work should explore how the resilience profiles measured by this scale relate to broader organisational outcomes, including staff retention, job satisfaction and the effectiveness of workforce support strategies.

Despite its contributions, this study had several limitations. First, the sample was limited to NHs in Korea, which may restrict the generalisability of our findings to other regions and countries. However, as the core challenges of NH staff are common across many countries, these findings may be relevant to similar care settings. Second, although the scale was rigorously validated using cross‐sectional data, longitudinal studies are required to evaluate its predictive validity and sensitivity to changes over time.

## 5 Conclusion

The resilience scale developed in this study is grounded in the lived experiences and contextual realities of the NH nursing staff. Through rigorous item generation and psychometric validation, the scale captured the internal and external dimensions of resilience that are uniquely relevant to NH settings. The four‐factor structure identified reflects the multifaceted nature of resilience among staff members who frequently face emotional strain, ethical dilemmas and resource limitations. Based on these findings, the scale provides a solid foundation for future research that seeks to evaluate, support and enhance NH staff resilience through targeted interventions. This scale is expected to contribute meaningfully to advancing evidence‐based approaches to staff development and workforce sustainability in geriatric care environments.

## Author Contributions

All authors have made a significant contribution to the works described, sufficient to warrant being listed within the authorship list and have been involved in the drafting and development of this final manuscript.

## Funding

This study was supported by a National Research Foundation of Korea grant funded by the Korea government (NRF‐2021R1I1A1A01048956). Additionally, this work was supported by the Soonchunhyang University Research Fund.

## Ethics Statement

This study was approved by the Institutional Review Board (IRB) of the Soonchunhyang University [No. 1040875–202,212‐SB‐133] before the start of the study and followed the principles specified in the Declaration of Helsinki. The researchers fully explained the purpose and method of the study to each participant before the start of the study and obtained informed consent.

## Conflicts of Interest

The authors declare no conflicts of interest.

## Data Availability

The data that support the findings of this study are available on request from the corresponding author. The data are not publicly available due to privacy or ethical restrictions.
